# Characteristics and plasticity of electrical synaptic transmission

**DOI:** 10.1186/s12860-016-0091-y

**Published:** 2016-05-24

**Authors:** Sebastian Curti, John O’Brien

**Affiliations:** Departamento de Fisiología, Facultad de Medicina, Universidad de la República, Montevideo, Uruguay; Department of Ophthalmology & Visual Science, University of Texas Health Science Center, Houston, TX USA

**Keywords:** Connexin 36, Mauthner cell, MesV neuron, Amacrine cell, Photoreceptor

## Abstract

Electrical synapses are an omnipresent feature of nervous systems, from the simple nerve nets of cnidarians to complex brains of mammals. Formed by gap junction channels between neurons, electrical synapses allow direct transmission of voltage signals between coupled cells. The relative simplicity of this arrangement belies the sophistication of these synapses. Coupling via electrical synapses can be regulated by a variety of mechanisms on times scales ranging from milliseconds to days, and active properties of the coupled neurons can impart emergent properties such as signal amplification, phase shifts and frequency-selective transmission. This article reviews the biophysical characteristics of electrical synapses and some of the core mechanisms that control their plasticity in the vertebrate central nervous system.

## Background

Organization of neurons into networks is a defining feature of a nervous system. Networks are essential for most complex computations and all conversions of sensory input to functional output. This network organization is accomplished by synapses, which provide the modes of communication between neurons. In all nervous systems, changes in synaptic strength are a fundamental tool to modify the network for a specific task, to emphasize a specific input or output, and to learn.

Two structurally and functionally different types of synapses, chemical and electrical, carry the burden of communication between neurons. Chemical synapses, with separate complex presynaptic and postsynaptic elements, have long been understood to be plastic, undergoing changes that strengthen or weaken the synapse under certain conditions. Gap junction-mediated electrical synapses are structurally simpler, giving rise to the misconception that they are also functionally simple. However, electrical synapses have been found to have great latitude for plasticity, contributing in many ways to the modification of network computations essential to optimize nervous system function. This review will briefly introduce electrical synapses and summarize the plastic mechanisms used to control neuronal coupling in order to optimize network functions.

### Properties of electrical synaptic transmission

**Fig. 1:**
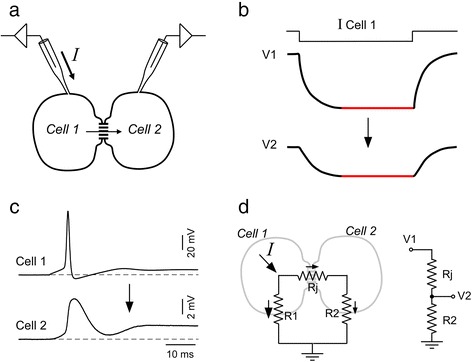
Basic properties of electrical coupling. **a** Schematic drawing of experimental design for study electrophysiological properties of electrical synapses showing simultaneous intracellular recordings using the dual whole cell patch clamp technique applied to a pair of coupled cells. **b** When a hyperpolarizing current pulse is injected to cell 1 (I Cell 1) a voltage deflection is produced in that cell (V1) and also in the cell 2 (V2), although voltage change in the later is of smaller amplitude. Traces are representative drawings. **c** An action potential in one cell (cell 1) of an electrically coupled pair produces a coupling potential or spikelet in the other cell (cell 2), which present a much slower time course compared to the presynaptic spike. **d** Left, Drawing shows the equivalent circuit for a pair of coupled cells during current injection into cell 1 (oblique arrow, I) where R1 and R2 represent the membrane resistance of cell 1 and cell 2 respectively and Rj represents the junctional resistance. For a voltage change at steady state (*red* portion of traces in B) the membrane capacitance is fully charged and current is only resistive. Smaller arrows indicate the direction of current flow in the circuit. Right, Circuit representing the voltage divider constituted by the junctional resistance (Rj) connected in series to the membrane resistance of the postsynaptic cell (R2). Input voltage is the membrane voltage change in the presynaptic cell (cell 1, V1), whereas the output voltage of the divider is the membrane voltage change in the postsynaptic cell (cell 2, V2)

Gap junctions are composed of aggregates of intercellular channels that connect the cytoplasm of two cells, constituting a pathway for the diffusion of small intracellular solutes between cells [1, 2]. Besides this chemical coupling, gap junctions support electrical coupling based on their ability to allow the movement of ions, thus representing a low resistance pathway for the direct flow of electrical current between cells (Fig. 1a, b). Because gap junction communication occurs without the involvement of any intermediary messenger as in chemical synapses, they provide a fast mechanism for intercellular synaptic transmission.

Beyond the first description in the motor giant synapse of the crayfish [3, 4], electrical transmission has been established in the nervous systems of many phyla, from primitive animals like jellyfish to more evolved ones like mammals [5]. In the mammalian brain electrotonic coupling between neurons has been identified in almost every structure including the neocortex, hippocampus, inferior olivary nucleus, cerebellar cortex, trigeminal mesencephalic nucleus, vestibular nucleus, hypothalamus, the spinal cord and the retina among others (for review see [6]).

In many cases these junctions behave as simple ohmic resistors through which current flow is determined by the difference in membrane voltage of coupled cells (transjunctional voltage) and the resistance of the junction. As such they support bi-directional communication and tend to equalize the membrane potentials of coupled cells. This means that activation of any cell of a coupled pair will produce a comparable attenuated potential (the coupling potential or spikelet) in the other cell (Fig. 1c). These characteristics of gap junction mediated transmission determine two distinctive physiological properties of electrical synapses: high speed and sign conservation. Both of these characteristics may promote the synchronic activation of neuronal ensembles. However, beyond these two well-established and classical roles, electrical coupling in conjunction with properties of the non-junctional membrane of neurons provides mechanisms for more complex operations like inhibition, amplification and frequency selective transmission.

### Determinants of the strength of electrical synapses

In most cases, electrical synapses can be considered to function as a simple resistance between two coupled neurons. Consequently, the degree to which a neuron is coupled to another can be described by the electrical influence a voltage change in one neuron has on its coupled neighbor, i.e. the coupling coefficient (C):

1 $$ C=\frac{V_2}{V_1} $$

where V_1_ is the voltage of the “driver” cell and V_2_ is the voltage of the “follower” cell. From this relationship it is evident that coupling potentials present the same sign as presynaptic signals but are smaller in amplitude (Fig. 1b). In the absence of voltage dependent mechanisms in the postsynaptic cell this coefficient varies between 0 and 1, and the bigger its value the stronger the degree to which two cells are electrically coupled.

For a voltage change at steady state the simplest electrical representation of two cells connected by a gap junction is the circuit depicted in the left panel of Fig. 1d, where Rj represents the junctional resistance, and R1 and R2 the membrane resistance of coupled cells [7]. Current injected in cell 1 present two parallel pathways to flow, one through R1 and the other involving Rj and R2, thus producing a voltage change in both the presynaptic cell (V1) and in the postsynaptic cell (V2). On the other hand, because Rj and R2 are connected in series they constitute a voltage divider or attenuator; that is, a simple circuit where the input voltage is split among the two components in a proportional fashion according to the value of their resistances, being the input voltage V1 and the output V2 (Fig. 1d, right panel). In a voltage divider the output voltage depends on the input voltage according the following equation [8]:

2 $$ V2=V1 \times \frac{R2}{R2+Rj} $$

From this equation

3 $$ \frac{V2}{V1} = \frac{R2}{R2+Rj},\  and\ as\ C = \frac{V2}{V1}\  then\ C = \frac{R2}{R2+Rj} $$

From the above analysis it can be concluded that the coupling coefficient depends both on the junctional resistance and the membrane (non-junctional) resistance of the second postsynaptic cell [7]. However, the strength of electrical transmission does not depend on the absolute value of any of these resistances but instead on the relationship between them (see below). While the junctional resistance depends on the properties of intercellular gap junction channels, the membrane resistance depends on the number of channels of the non-junctional membrane open at resting potential and is a major determinant of the input resistance of neurons and hence of the way they respond to synaptic inputs.

### Plasticity of electrical synapses

Given that the strength of coupling between neurons depends on dynamic factors such as the resistance of the gap junction and the membrane resistance of the postsynaptic cell, it should be clear that coupling also changes dynamically. Indeed, all aspects that control electrical synaptic strength can change over a wide variety of time scales ranging from milliseconds to days, with different mechanisms participating at different time scales. These mechanisms will be treated separately below.

### Changes in conductance of electrical synapses

#### Voltage gating of connexin channels

Like many other membrane ion channels, gap junction channels display some degree of voltage sensitivity [1, 9]. Voltage gating of connexin channels results in shifts to a low conductance state or subconductance state at the level of the individual channel [9]. Dynamic voltage gating has been observed to occur during cardiac myocyte action potentials [10] and contributes to the waveform and propagation of the action potential through the syncytium. This gating behavior was attributed largely to Cx43 channels, which are the dominant connexin in cardiac myocytes.

In contrast to cardiac gap junctions, gap junction channels formed by Cx36, the main synaptic connexin of the mammalian brain, present a weak voltage-dependency. In fact, junctional conductance is nearly insensitive to transjunctional voltage up to ±30 mV and declines gradually to ~60 % over a 90 mV range. Moreover, the time course of the underlying gating process requires hundreds of milliseconds to seconds to reach the steady state [11–13]. While gating processes of gap junction channels are able to produce a substantial modification of the junctional conductance, these changes occur in time scales several orders of magnitude larger than that of single spikes and synaptic potentials, the main source of coupling potentials in physiological conditions. Thus electrical synapses composed of Cx36 are unlikely to be susceptible to voltage gating during normal neuronal activity.

Other connexins that form electrical synapses in the vertebrate nervous systems exhibit more robust voltage gating. Cx45, which is present in a small number of electrical synapses, is particularly sensitive to transjunctional voltage [14, 15], with half maximal reduction of the voltage-sensitive conductance at 13.4 mV in the steady state. While voltage gating of connexin channels is driven largely by the “fast gate” [9], the kinetics of this mechanism are nonetheless somewhat slow and unlikely to have a large impact on channel conductance during a neuronal action potential. However, gating is likely to occur in neurons that use sustained, graded voltage signaling such as retinal bipolar cells, some of which do use Cx45 in electrical synapses [16–18]. The impact of any such changes on electrical signaling is unknown.

#### Phosphorylation and dephosphorylation of channels

Very significant changes in the overall conductance of gap junction channels that form electrical synapses occur through signaling pathways that result in phosphorylation or dephosphorylation of connexins. Studies of retinal horizontal cells have shown that catecholamines, dopamine in particular, reduce the receptive field size and tracer coupling [19–22]. These effects were shown to result from activation of a D1 dopamine receptor that elevated intracellular cAMP via adenylyl cyclase activity [23–25], and depended on activation of protein kinase A [26]. The reduced electrical coupling in fish horizontal cells resulted from a reduction in the open probability of the gap junction channels without a change in unitary conductance [27]. The horizontal cells in fish contain several connexins: Cx55.5, Cx52.6, and Cx52.9 have all been identified in zebrafish [28–30]. It is not clear which, if any, of these contribute to the plasticity that has been observed in horizontal cells from the fish species studied physiologically.

The vast majority of electrical synapses in the mammalian central nervous system utilize Cx36 (homologous to Cx35 in non-mammalian vertebrates). A number of in vitro studies have shown that electrical or tracer coupling via this connexin is regulated by phosphorylation driven by cAMP/PKA [31, 32], nitric oxide/PKG [33], and Ca^2+^/CaMKII signaling pathways [34, 35], with a few conserved phosphorylation sites being key regulators of coupling. The biophysical basis of changes in macroscopic coupling has not been elucidated but changes in channel open probability, based upon changes in mean open time, have been suggested as the mechanism of plasticity [35].

A number of studies have revealed that Cx36 phosphorylation state changes with conditions that change coupling and is an accurate, and essentially linear, predictor of coupling as assessed by tracer transfer [36–39]. In retinal neurons, phosphorylation-dependent changes in coupling are driven by light adaptation [38–40] and/or circadian rhythms [41–43]. The signaling pathways that control these changes have been studied in detail in photoreceptor and AII amacrine cells in recent years, revealing a common theme of regulation by well-defined opposing signaling pathways.

**Fig. 2:**
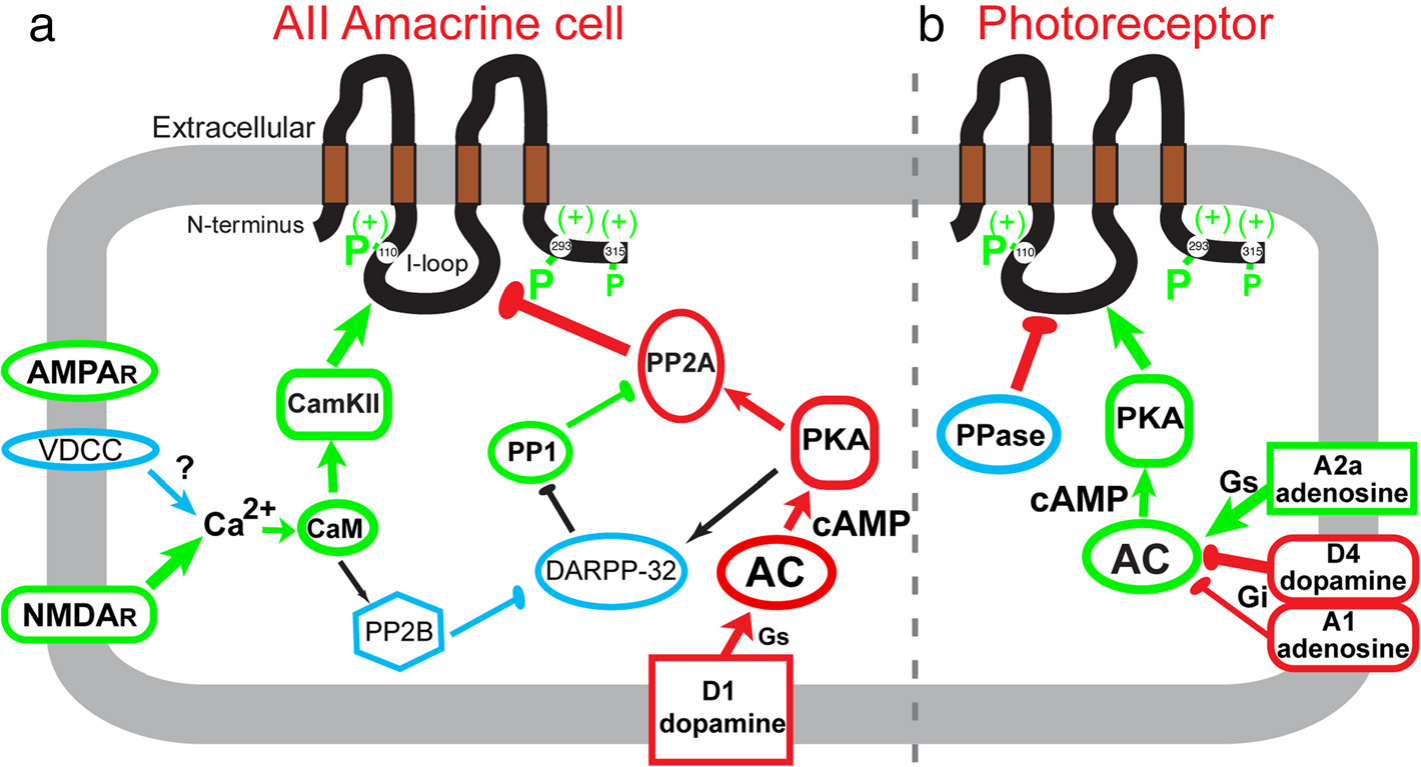
Signaling pathways that control coupling in two types of retinal neuron. Coupling through Cx36 gap junctions is regulated by Cx36 phosphorylation through an order of magnitude dynamic range. Phosphorylation enhances coupling and pathways that promote Cx36 phosphorylation are colored green in this diagram while those that reduce phosphorylation are colored red. Elements colored blue are hypothesized to play a role but have not been specifically demonstrated. **a** Retinal AII amacrine cell coupling is increased by Cam Kinase II phosphorylation driven by Ca^2+^ influx through non-synaptic NMDA-type glutamate receptors. This process depends on spillover glutamate derived from bipolar cells and is enhanced by activation of synaptic AMPA-type glutamate receptors that depolarize the cell. Reduction of Cx36 phosphorylation is driven by an independent pathway in which activation of D1 dopamine receptors increases adenylyl cyclase activity, activating protein kinase A, which in turn activates protein phosphatase 2A. Protein phosphatase 1 suppresses this pathway. Both pathways are activated by light, but with different thresholds, leading to an inverted U-shaped light adaptation curve. **b** Photoreceptor coupling is enhanced by Cx36 phosphorylation driven directly by protein kinase A activity under control of adenylyl cyclase (AC). AC activity is in turn controlled by an intricate set of G-protein coupled receptors regulated by circadian time and light adaptation. Darkness during the night phase increases extracellular adenosine such that activation of A2a adenosine receptors dominates signaling and activates AC. Light adaptation or subjective daytime result in reduced extracellular adenosine and increased dopamine secretion such that activation of dopamine D4 receptors dominates signaling to suppress AC activity. A1 adenosine receptors supplement this effect. The opposing signaling pathways routed through a common effector impart a steep monophasic character to the light adaptation and circadian control of coupling in this neural network

A role for dopamine D2-like receptors in controlling rod to cone photoreceptor coupling has been known for some time [44, 45]. In rodents, this is actually a D4 receptor [39, 46], which inhibits adenylyl cyclase via Gi and reduces cAMP level. Phosphorylation of Cx36 is controlled by protein kinase A (PKA) activity, changing in response to alteration of cytoplasmic cAMP [38, 39, 47] (Fig. 2). In both mouse and zebrafish, the action of the dopamine D4 receptor is opposed by the action of a Gs-coupled adenosine A2a receptor [39, 47]. Secreted dopamine and extracellular adenosine levels vary in retina in opposite phase and are both regulated by circadian rhythms [48]: dopamine is high in the daytime or subjective day while adenosine is high in nighttime or subjective night. Li et al. [47] have recently found that the Adenosine A1 receptor is also present. The Gi-coupled A1 receptor has higher affinity for adenosine than does the A2a and is activated in the daytime by the lower extracellular adenosine level that remains. This A1 receptor activation reinforces the inhibitory action of the dopamine D4 receptor on adenylyl cyclase, strongly suppressing Cx36 phosphorylation and photoreceptor coupling in the daytime [47]. Since all three receptors act on the same target, adenylyl cyclase, the regulation of Cx36 phosphorylation and photoreceptor coupling is a steep biphasic function that keeps coupling minimal during the daytime (Fig. 2).

In retinal AII amacrine cells, plasticity of electrical coupling has been recognized for nearly 25 years [49]. This plasticity is driven by light, with a biphasic pattern showing very low coupling in prolonged dark-adapted conditions, high coupling with low-intensity illumination, and low coupling again with bright illumination [50, 51]. The bright light-driven reduction in coupling is mediated by dopamine, with dopamine D1 receptors increasing adenylyl cyclase activity and enhancing protein kinase A activity [49, 52]. AII amacrine cells use Cx36 [53], and the suppression of coupling by protein kinase A activity is inconsistent with the positive effect that protein kinase A activity has on photoreceptor coupling mediated by Cx36 [38, 39]. This contradiction was resolved by Kothmann et al. [37], who demonstrated that PKA activity in turn activated protein phosphatase 2A to drive dephosphorylation of Cx36 in AII amacrine cells (Fig. 2), resulting in uncoupling.

The ascending leg of the AII amacrine cell’s biphasic light adaptation curve depends on the activity of glutamatergic On pathway bipolar cells, which are first-order excitatory interneurons postsynaptic to photoreceptors. Like other forms of activity-dependent potentiation, enhancement of AII amacrine cell coupling results from activation of NMDA receptors, Ca^2+^ influx, and activation of Cam Kinase II, which phosphorylates Cx36 [40]. The NMDA receptors on AII amacrine cells are non-synaptic and are closely associated with Cx36 [40], so their activation depends on spillover glutamate. This most likely comes from rod bipolar cells, which are presynaptic to the AII amacrine cell, but may also come from cone On bipolar cells that are nearby. Because the signaling pathways in AII amacrine cells that phosphorylate and dephosphorylate Cx36 are independent (Fig. 2) and have different illumination thresholds, the light adaptation curve of the AII amacrine cell shows its characteristic biphasic pattern.

The activity-dependent potentiation of AII amacrine cell electrical synapses resembles that originally described in the mixed synapse of auditory VIIIth nerve club endings onto Mauthner cells in the goldfish [54, 55]. Plasticity in the Mauthner cell differs in that the NMDA receptors that provide the Ca^2+^ signal are synaptic and require high-frequency stimulation to potentiate. A similar form of plasticity dependent upon non-synaptic NMDA receptors has also been described recently in rat inferior olive neurons [56].

A variety of other signaling pathways have been found to modulate electrical synapses. In interneurons of the thalamic reticular nucleus (TRN), excitatory input depresses electrical synapses through activation of metabotropic glutamate receptors (mGluRs) [57]. This signaling has been explored in detail recently. Both group I and group II mGluRs modulate coupling, but with opposite effects [58]. The dominant effect appears to be through activation of Group I mGluRs, which produce long-term depression by activation of a Gs signaling pathway, stimulating adenylyl cyclase and activating PKA. However, selective activation of the group II receptor mGluR3 promotes long-term potentiation through activation of Gi/o [58]. This shares the same pathway, routing ultimately through PKA activity. Since TRN neurons employ Cx36 [59], through which electrical coupling is increased by phosphorylation [35, 37–39], this signaling mechanism must include a PKA-activated phosphatase to reduce Cx36 phosphorylation upon PKA activation in a manner similar to that in retinal AII amacrine cells.

Histamine H1 and H2 receptors have been found to modulate coupling among various populations of neurons in the supraoptic nucleus [60, 61]. H2 receptors signal through adenylyl cyclase, but H1 receptors instead activate NO synthase, signaling through nitric oxide, guanylyl cyclase, and protein kinase G. A potentially similar nitric oxide-driven signaling pathway also selectively regulates the heterologous electrical synapses between retinal AII amacrine cells and cone On bipolar cells [52]. Thus it is apparent that a wide variety of signaling pathways have been employed to regulate electrical synaptic strength via connexin phosphorylation and dephosphorylation in different neurons throughout the central nervous system.

#### Changes in number of channels

Changes in the expression level of connexins provide a mechanism to alter coupling over time scales of hours to weeks. Such changes are most prominent in development. Electrical coupling in most areas of the vertebrate CNS tends to increase to high levels in early phases of development, and then reduce again [62–64]. One study found that activation of group II mGLuRs was responsible for the developmental increase of coupling, acting both through transcriptional and post-transcriptional mechanisms [65].

A surprisingly similar increase in neuronal coupling is also seen following various types of injury [66]. Ischemic injuries result in an increase in neuronal coupling and the level of Cx36 protein, without an apparent increase in transcript level [67, 68]. This has been attributed to group II mGluR activation, as was the developmental increase, with dependence on a cAMP/PKA signaling pathway [68]. Traumatic injuries [69, 70] and seizures [71, 72] also result in increases of neuronal coupling, although these insults lead to increases in Cx36 transcript level. In these contexts, alteration in the expression level of connexins that form electrical synapses are important factors in long term changes in neuronal coupling.

Electrical coupling of mature neurons is critically dependent on maintenance of a steady state population of gap junction proteins. A recent study showed that electrical coupling in goldfish Mauthner cell mixed synapses was reduced within a few minutes if perturbed by peptides that disrupted stabilizing interactions of Cx35 with scaffolding proteins or blocked SNARE-mediated trafficking of new Cx35 [73]. Another study found circadian regulation of Cx36 transcript and protein levels in photoreceptors [74]. These studies reveal that electrical synapses are dynamic structures whose channels are turned over actively, suggesting that regulated trafficking of connexons may contribute to the modification of gap junctional conductance.

### The role of the passive properties of the postsynaptic cell

#### The membrane resistance of the postsynaptic cell

As previously mentioned electrical coupling depends on both the resistance of the gap junction and the membrane resistance of the postsynaptic cell. In fact, while changes of the gap junction resistance due to modifications of the single channel conductance or the number of intercellular channels might produce significant changes in the coupling coefficient, modifications of the postsynaptic membrane can also underlie significant and highly dynamic changes in the strength of electrical coupling representing an additional point of regulation. The fact that the junctional resistance (Rj) and the membrane resistance of the postsynaptic cell (R2) constitute a voltage divider (Fig. 1d) implies that when Rj is big compared to R2 most of the input voltage will drop across Rj and only a minor fraction across R2 meaning a modest voltage change in the postsynaptic cell which corresponds to a low coupling coefficient. In contrast, if R2 is big compared to Rj a correspondingly big fraction of the input voltage (V1) will appear across the membrane of the postsynaptic cell (V2). A large voltage drop across R2 corresponds to a large coupling coefficient meaning that cells are strongly coupled. This dependency of coupling coefficient on the input resistance of the postsynaptic cell determines the directionality of transmission when electrical coupling occurs between cells of dissimilar input resistances. In fact, electrical transmission will be more efficient from the lower input resistance to the higher input resistance cell in comparison to the opposite direction. Therefore, despite of the presence of non-rectifying contacts, symmetrical communication will occur only when connected cells present similar input resistances. Hence, the directionality of electrical transmission imposed by asymmetry of passive properties of connected cells might be a key determinant of the flow of information within neural circuits.

#### Modification of passive membrane properties by synaptic inputs

Interestingly, modifications of the membrane resistance (Rm) of coupled cells due to nearby chemically mediated synaptic actions can significantly modulate the strength of electrical coupling in a highly dynamical fashion [5, 75]. In fact, as these synaptic actions usually involve changes of membrane permeability to different ion species, they are accompanied by corresponding changes in membrane resistance of the postsynaptic cell and hence of the strength of electrical coupling. Typically, excitatory synaptic actions are mediated either by increased membrane permeability to Na^+^ and K^+^ (decreased Rm) or by a decreased permeability to K^+^ (increased Rm). Usually, synaptic actions are defined by the sign of its effect on membrane potential of the postsynaptic cell (depolarization versus hyperpolarization). What is remarkable is that although both synaptic actions are depolarizing shifts of membrane voltage they have opposite effects on the efficacy of electrical transmission. Whereas synaptic actions involving an increase in Rm enhance the strength of coupling, a reduction in Rm elicits an uncoupling of electrically connected cells [76]. A similar shunting effect by nearby GABAergic inputs has been proposed to underlie decoupling in pairs of inferior olivary neurons [77, 78]. These results indicate that the membrane resistance of the postsynaptic cell is a key element for regulating electrical coupling, being as important as the junctional resistance. This means that changes in the efficacy of electrical synapses might be accomplished through modification of either of these two resistances. Alternatively, when electrical coupling is expected to be constant in order to assure stable network function, changes in electrophysiological properties of coupled cells require corresponding changes of junctional resistance. In fact, concurrent changes of the junctional and membrane resistances of coupled cells in a homeostatic fashion has been proposed to underlie the stability of electrical coupling strength between neurons of the thalamic reticular nucleus during development [79].

#### The time constant of the postsynaptic cell

The time course of membrane voltage changes is dominated by the cell’s capacitance, which results from the ability of biological membranes to separate electrical charges. In fact, while a simple ohmic resistor responds to a step current with a similar voltage step, cells show voltage responses that rise and decay more slowly than the current step (Fig. 1b). This property of the membrane can be modeled by a resistor connected in parallel to a capacitor. The ability of this circuit to slow down changes in voltage results from the fact that a discharged capacitance offers no resistance to current flow, determining that at the beginning of the current step all current will flow through the capacitance and nothing through the resistance. As the capacitance gets charged it progressively develops more resistance to current and more current will flow through the resistance [80].

This circuit comprises a simple low pass filter for input currents characterized by its time constant. Indeed, the resistance of the gap junction connected in series to the parallel resistance and capacitance of the postsynaptic cell behaves as a low-pass filter determining that the high-frequency components of presynaptic signals are comparatively more attenuated. That is, slow fluctuations of membrane voltage pass more effectively between cells than do fast signals [7, 81]. This is a characteristic property of electrical transmission and underlies the fact that coupling potentials present a slower time course in comparison to the presynaptic signals that generated them (Fig. 1c). As a result of this property, a delay of postsynaptic responses is introduced with respect to the presynaptic signals. This property of low-pass filters, known as phase lag, represents the synaptic delay of electrical synapses. Although current begins to flow across the junction without delay, time is required for charging the postsynaptic capacitance to a significant level to generate a detectable voltage change above the noise level [81].

**Fig. 3:**
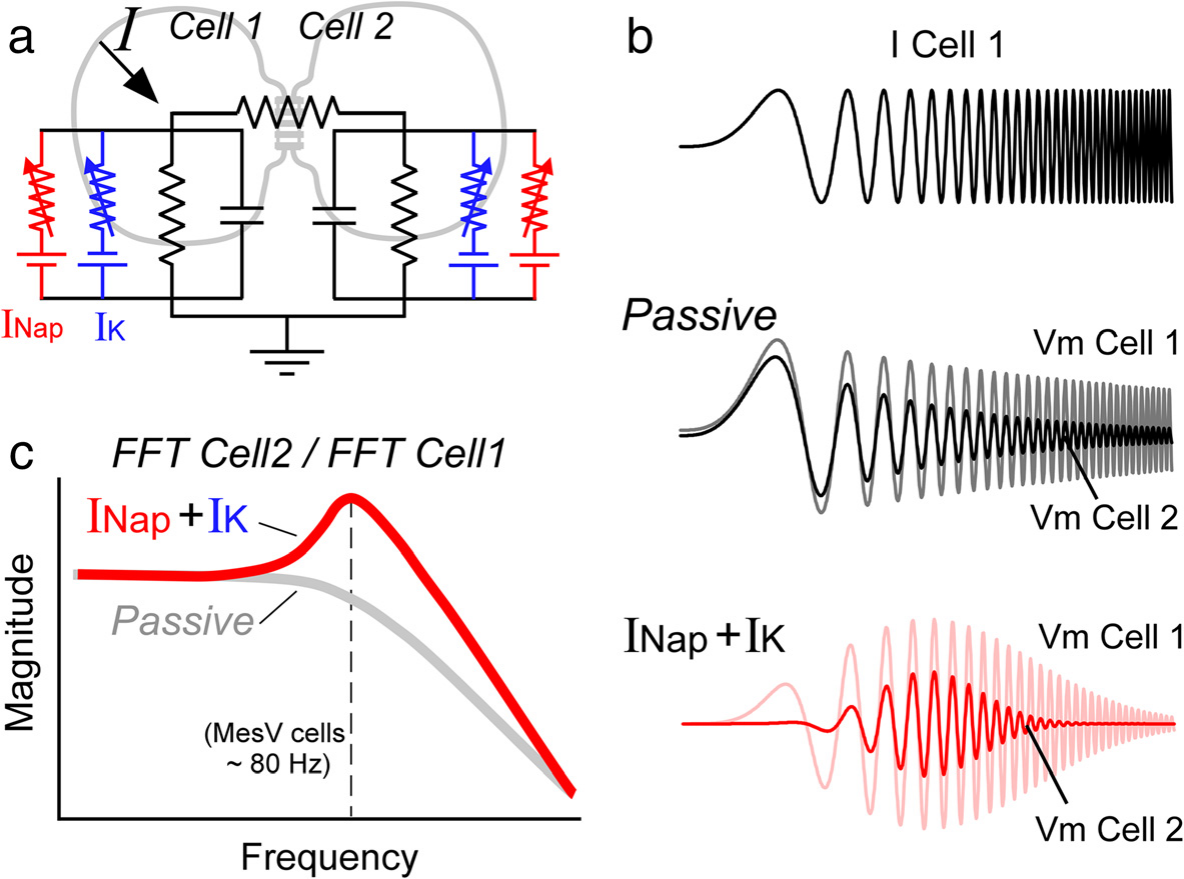
Frequency selectivity of electrical transmission. **a** Equivalent circuit of a pair of coupled cells including the passive elements (resistance and capacitance, *black*) and active voltage-dependent conductances (INap and IK) represented as a variable resistor in series to an EMF. **b** Top panel, Sinusoidal current waveform of increasing frequency (ZAP protocol) is injected into cell 1 (I Cell 1) in order to test the frequency-dependent properties of electrical transmission between coupled cells. Middle, Superimposed are depicted the voltage membrane responses of the presynaptic cell (Vm Cell 1) and of the postsynaptic cell (Vm Cell 2) for a pair of coupled cells which include only passive elements (RC circuit, black elements in circuit in A). Both responses are characteristics of a low-pass filter where amplitude of membrane response decreases monotonically as sinusoidal frequency increases. Bottom, By contrast, when cells present passive and active voltage-dependent currents (IK and INap) membrane responses present certain frequency selectivity where signals close to the characteristic frequency are of bigger amplitude compared to signals whose frequency lie far from this value. **c** Schematic plot of the frequency transfer characteristics of electrical transmission calculated as the ratio of the FFT of the postsynaptic membrane response over the FFT of the presynaptic membrane response depicted in B, for a pair of passive cells (*gray trace*) and for a pair of cells which also present resonant and amplifying currents (IK and INap respectively). Whereas transfer function when cells present only passive elements show the typical profile of a low-pass filter (*gray trace*), the presence of voltage-dependent currents determines that transmission of signals near the characteristic frequency (*vertical dashed line*) is less attenuated, determining a maximum in the function (*red trace*). Traces are representative drawings

Early descriptions of electrical synapses in invertebrates already proposed that these contacts present low-pass filtering characteristics [4, 82, 83]. More recently, filtering characteristics of electrical transmission between mammalian central neurons have been demonstrated by using dual whole cell patch recordings and injecting sinusoidal currents of different frequencies (Fig. 3b). Under these experimental conditions, coupling coefficients and phase lag were determined as a function of sinusoidal frequency. This experimental approach in different cell types like GABAergic interneurons of the neocortex [84–86], neurons of the thalamic reticular nucleus [59], Golgi cells of the cerebellum [87], retinal AII amacrine cells [88] among others, confirmed that electrical transmission presents low-pass filter characteristics, allowing the passage of low frequency signals but strongly attenuating and delaying signals of higher frequency [6].

This property of electrical synapses determines that slow potential changes (typically subthreshold) are preferentially transmitted over action potentials, endowing electrical synapses with the ability to transmit different information than the spikes transmitted via chemical synapses. For instance, in cell types where action potentials are followed by a large and prolonged after-hyperpolarization (AHP) due to the delayed activation of a voltage- and/or Ca^++^ dependent K^+^ current, coupling potentials tend to be predominantly hyperpolarizing events. This phenomenon results from the low-pass filter properties of electrical transmission. In fact, because the high-frequency components of the fast presynaptic action potential are more attenuated than the slow AHP, the coupling potential results in a net hyperpolarizing signal, inhibiting neural activity rather than promoting activation of the postsynaptic neuron [85, 87, 89]. In the cerebellar cortex, this effect has been involved in the desynchronization of the population of Golgi cells due to sparse depolarizing synaptic inputs [90].

### The role of the active membrane properties of the postsynaptic cell

#### Electrophysiological properties of neurons

In addition to the passive membrane properties (those that are linear with respect to the membrane voltage), excitable cells like neurons present active membrane properties, which are highly non-linear mechanisms due to complex time and voltage dependent processes. The most remarkable outcome of the active membrane properties is the action potential generation underlain by the classical Na^+^ and K^+^ conductances described by Hodgkin and Huxley in the squid axon [91]. Despite these spike-generating mechanisms which allow neurons to communicate over long distances in a non-decremental fashion, excitable cells usually present a large variety of subthreshold active properties. These active mechanisms along with the passive properties establish the way neurons integrate spatially and temporally distributed synaptic inputs, and how these inputs are translated or encoded into a time series of action potentials. The active membrane properties of neurons depend on the kind, density and distribution of voltage operated ion channels in the surface membrane of the different cellular compartments. Central neurons present a rich repertoire of voltage operated membrane ion channels that endow them with powerful encoding capabilities represented by the ability to transform their inputs into complex firing patterns. Indeed, neurons express tens of different voltage operated membrane conductances according to their ion selectivity, voltage range of activation, kinetics, presence of inactivation, and modulation by intracellular second messengers giving rise to a wide variety of electrophysiological phenotypes [92–95].

#### Voltage dependency of coupling potential

Despite the limited voltage gating of connexin intercellular channels imposed by its slow kinetics, electrical coupling between neurons might present marked voltage-dependency. However, this phenomenon does not represent a voltage dependent property of the gap junctions but instead are supported by the active properties of the non-junctional membrane of the postsynaptic cell. For instance, in fish a pair of gigantic command neurons, the Mauthner cells, which are responsible for the initiation of escape responses, are contacted by a special class of auditory afferents through mixed electrical and chemical synaptic contacts [96]. These electrical contacts not only allow the forward transmission of signals (from afferents to the Mauthner cell), but also support retrograde transmission by allowing the spread of dendritic postsynaptic depolarizations to the presynaptic afferents. Moreover, retrograde coupling potentials in the afferents present a marked voltage dependency. In fact, depolarization of the membrane potential of these afferents evokes a dramatic increase in coupling potential amplitude, eventually enough to activate them, and hence supporting a mechanism of lateral excitation whereby the sound-evoked activation of some afferents can recruit more afferents to reinforce the synaptic action on Mauthner cells [97, 98]. This amplifying mechanism is blocked by extracellular application of tetrodotoxin (TTX) or intracellular injection of QX-314, strongly suggesting the involvement of a Na^+^ current. Additionally, its subthreshold voltage range of activation, among other properties, indicates that the persistent sodium current (INap) of these afferents is the underlying mechanism of this amplification [98].

The INap is a non-inactivating fraction of the Na^+^ current, which activates at subthreshold membrane voltages and is particularly well suited to perform such amplification because of its rapid kinetics and subthreshold membrane voltage range of activation. In the mammalian brain similar amplifying mechanisms of coupling potentials involving Na^+^ currents have been described in the mesencephalic trigeminal (MesV) nucleus of the rat [99]. This cell population is coupled mostly in pairs and activation of one neuron of an electrically coupled pair produces a spikelet in the postsynaptic cell (Fig. 1c). This coupling potential critically depends on the membrane potential, being enhanced by depolarization of the postsynaptic cell and eventually triggering an action potential in this cell. This spikelet exhibits a positive correlation with the membrane potential of the postsynaptic cell, and because of its voltage range of activation and sensitivity to sodium channel blockers it represent the activation of a persistent sodium current [99]. Similar amplifying mechanism has been proposed in the cerebellar cortex [87, 100] and the thalamic reticular nucleus [101]. Thus, the INap endows electrical coupling with voltage-dependent amplification, suggesting a relevant contribution of active membrane conductances in regulating the efficacy of electrical transmission between neurons. Moreover, as such amplification of electrotonic potentials might be enough to recruit the postsynaptic cell, it tends to synchronize the activity of networks of neurons, emphasizing the role of active conductances in the dynamics of networks of electrically coupled neurons.

#### Frequency selective transmission

Most typically electrical transmission between neurons possesses low-pass filter properties imposed by the RC circuit of the postsynaptic cell. In contrast, electrical coupling between MesV neurons show band-pass filter properties where signals with frequencies in the range of 50 to 80 Hz are preferentially transmitted, even better than DC signals (Fig. 3) [99]. Accordingly, transmission of spikes through these contacts is significantly more efficient than in electrical contacts between FS or LTS interneurons of the neocortex, whose frequency transfer resembles a low-pass filter [86]. This suggests that electrical transmission between MesV neurons is well suited for the transmission of action potentials, which most probably constitute the main signal source for coupling and promotes the synchronic activation of pairs of MesV neurons [99].

This frequency selectivity or band-pass characteristics results from the resonant properties of MesV neurons. Resonance is a property that enables neurons to discriminate between its inputs on the basis of their frequency content, so that synaptic inputs with frequency content close to the resonant frequency will produce the largest responses. Resonance arises from the interplay of two mechanisms with specific frequency-domain properties: the passive and the active membrane properties. As previously discussed, passive properties due to the capacitance in parallel with the conductance of the membrane act as a low-pass filter (whose cutoff frequency is set by the time constant of the membrane), attenuating responses to inputs with high frequency content. On the other hand, certain voltage-dependent conductances that actively oppose changes in membrane voltage, like K^+^ currents, might confer high-pass filter properties (whose cutoff frequency is set by its activation time constant), thus attenuating responses to inputs with low frequency content. While these two mechanisms with opposite filter properties are present in almost every neuronal type, as low-pass filtering due to the RC circuit is a basic property of biological membranes and K^+^ currents are ubiquitous conductances, not every neuron expresses resonance. In fact, to produce resonance K^+^ current must activate slowly compared to the membrane time constant. Thus, the combination of these two mechanisms with appropriate cutoff frequencies creates a band-pass or resonant filter, capable of rejecting inputs whose frequencies lie outside this band [102].

Although the combination of these two mechanisms sets the frequency of resonance, its expression typically depends on the activation of amplifying currents. Such currents are essentially the inverse of resonant currents, that is, they amplify voltage changes and activate quickly relative to the membrane time constant. The persistent Na^+^ current is an example of such an amplifying current whose interaction with resonant currents enhances resonance. This frequency preference endows neurons with the ability to generate spontaneous membrane voltage oscillations and repetitive discharges, or to respond best to inputs within a narrow frequency window [102]. In the context of electrical synaptic transmission, resonance will determine that signals with frequency content near the resonant frequency will be more readily transmitted than other signals, even better than DC signals, promoting the transmission of signals of biological relevance (Fig. 3).

MesV neurons are endowed with a rich repertoire of voltage-gated membrane conductances, like the A-type K^+^ current (IA) and the INap supporting resonance, which results in the generation of membrane voltage subthreshold oscillations and repetitive discharges in the range of 50 to 100 Hz [103, 104]. Consistently, electrical transmission between MesV neurons exhibits band-pass filter characteristics instead of the classical low-pass filter properties [99]. In fact, the assessment of the filter properties by means of injecting frequency-modulated sine wave currents (ZAP protocols, Fig. 3b) and calculating the ratio of the Fast Fourier Transform (FFT) of the postsynaptic voltage changes over the FFT of the presynaptic voltage changes, showed a peak in the range of 50 – 100 Hz (Fig. 3c) [99]. Thus the frequency transfer function of electrical transmission between MesV neurons presents a maximum at frequencies near 80 Hz, indicating that transmission of electrical signals between MesV neurons exhibits some degree of frequency preference and therefore does not behave as a simple low-pass filter [99]. Consistent with the critical role of active membrane properties in determining frequency selective transmission at these electrical contacts, the addition of TTX (0.5 μM) to the extracellular solution results in a reduction of the amplitude of the transfer function, particularly for values around 50–80 Hz, indicating the participation of Na^+^ conductances. The subsequent addition of 4-AP (1 mM), a blocker of the A-type current among other K^+^ conductances, further modifies the transfer characteristics resembling now the properties of a simple low-pass filter (Fig. 3c). These voltage dependent conductances not only improve transmission in terms of the amplitude of postsynaptic signals, but also by reducing the phase lag between presynaptic and postsynaptic responses. Hence, while amplification increases the efficacy of synaptic transmission the mitigation of the phase lag at the same frequency range improves its accuracy, promoting the synchronic activation of pairs of coupled MesV neurons [99].

Therefore, the active membrane properties of neurons might play a critical role in synaptic electrical transmission by providing an extremely sensitive mechanism of voltage dependent amplification of electrical coupling potentials and endowing this modality of interneuronal communication with frequency selectivity. Moreover, modulation of voltage dependent conductances of the non-junctional membrane by the action of neurotransmitters represents a potential source of modulation of the efficacy of electrical transmission.

## Conclusions

In spite of the relative simplicity of the gap junction and the straightforward rules that govern electrical transmission, electrical synapses formed by gap junctions are far from simple. Dynamic processes affecting the resistance of the electrical synapse and the membrane resistance of the coupled cells can alter coupling on timescales ranging from milliseconds to days. Active membrane properties of the coupled cells can selectively enhance signals with certain frequency content, imparting band-pass filter properties to the coupled network. Combined these factors endow electrical synapses with a great deal of sophistication. With their high abundance and diverse roles in neural networks throughout the CNS, electrical synapses must be considered every bit as important as chemical synapses in the expression of neural plasticity.
